# Volumetric decrease of pancreas after abdominal irradiation, it is time to consider pancreas as an organ at risk for radiotherapy planning

**DOI:** 10.1186/s13014-018-1189-5

**Published:** 2018-12-03

**Authors:** Cengiz Gemici, Gokhan Yaprak, Sevim Ozdemir, Tamer Baysal, Ozgur Ozan Seseogullari, Hazan Ozyurt

**Affiliations:** 1 Department of Radiation Oncology, Dr. Lutfi Kirdar Kartal Education and Research Hospital, Istanbul, Turkey; 2 Department of Radiology, Dr. Lutfi Kirdar Kartal Education and Research Hospital, Istanbul, Turkey; 3 Department of Radiation Oncology, Biruni University Medicana International Hospital, Istanbul, Turkey

**Keywords:** Pancreas, Exocrine insufficiency, Endocrine insufficiency, Organ at risk, Radiotherapy planning

## Abstract

**Background:**

Volumetric shrinkage of normal tissues such as salivary glands, kidneys, hippocampus are observed after radiotherapy. We aimed to assess the alterations in pancreatic volume of patients who received abdominal radiotherapy and define pancreas as an organ at risk for radiation treatment planning.

**Material-methods:**

Forty-nine patients operated for gastric adenocarcinoma who received adjuvant abdominal radiotherapy were in the study group, 27 patients with early stage disease who did not need adjuvant treatment after surgery comprised the control group. An experienced radiologist contoured the pancreas of all the patients from computed tomographies imported to the planning system obtained either for radiation planning purpose or for follow-up after surgery. The same procedure was repeated one year later for both groups. Measured volume of the pancreas was expressed in cm^3^.

**Results:**

Mean pancreatic volumes were similar in both groups at the onset of the study, 51,34 ± 20,33 cm^3^, and 50,12 ± 23,75 cm^3^ in the irradiated and the control groups respectively (*p* = 0,63). One year later, mean pancreatic volumes were significantly decreased in each group; 22,48 ± 10,53 cm^3^, 44,18 ± 23,08 cm^3^ respectively, *p* < 0,001. However, the decrease in pancreatic volume was significantly more pronounced in the irradiated group in comparison to the control group, p < 0,001.

**Conclusion:**

Volumetric decrease in normal tissues after radiotherapy is responsible for loss of organ function and radiation related late side effects. Although pancreas is a radiation sensitive organ losing its volume and function after radiation exposure, it is not yet considered as an organ at risk for radiation treatment planning. Pancreas should be contoured as an organ at risk, dose-volume histogram for the organ should be created, and safe organ doses should be determined. This is the first study declaring pancreas as an organ at risk for radiation toxicity and the necessity of defining dose constraints for the organ.

## Background

Radiation related acute and especially late toxicity is a major concern and well defined for certain tissues and organs in radiation oncology practice [1, 2].

Volumetric changes and atrophy of organs at risk during and after irradiation is an important concern and is responsible for late effects of irradiation. The well-known organs in this respect are the salivary glands, kidneys, cerebral structures like hippocampus [3–7]. Parotid glands are the most studied organs among others, demonstrating a decrease of volume during and after radiotherapy [6–9]. Therefore, xerostomia as acute and late side effect is often encountered, and parotid glands are considered as the major risk organs for head and neck radiotherapy planning. Generally, the mean radiation doses to the parotids, kidneys and nowadays to the hippocampus are kept as low as possible to prevent late side effects like xerostomia, renal failure and cognitive functions. Mean radiation doses and dose constraints for these organs and tissues are very well defined for head and neck, abdominal and cranial radiotherapy. They are among the major organs at risk determining dose restrictions during treatment planning [1, 2].

Pancreas is a secretory organ which has resemblance to salivary glands having acinar and Langerhans cells with important exocrine and endocrine functions. Acinar cells both in the salivary glands and the pancreas secrete fluid and enzymes which are important for gastrointestinal system and digestion [10]. The two organs are clinically and functionally similar to each other [11]. Loss of exocrine function of the pancreas causes malabsorption and loss of endocrine function results in diabetes mellitus. While acinar cells are homogenously distributed throughout the pancreas, islets of Langerhans (beta-cells) are often located in the tail of the pancreas. This information may be useful for radiation planning.

In our previous study, we demonstrated that pancreatic tissue undergoes significant functional and structural changes after abdominal irradiation which is characterized by biochemical findings indicating diabetes mellitus risk and endocrine pancreatic insufficiency [12]. Furthermore, another recent study demonstrated loss of exocrine function of the organ after abdominal irradiation [13]. These studies confirm that pancreas loses its functional capacities after abdominal irradiation. However, pancreas is not yet considered as an organ at risk for radiotherapy planning and it is not contoured in order to define any dose constraints for treatment planning.

In this prospective study we aim to demonstrate that there is volumetric loss of organ after radiotherapy as observed in the salivary glands. Somehow, we can consider pancreas as the salivary gland of gastrointestinal tract. We hypothesize that the previously described endocrine and exocrine functional losses of the pancreas after abdominal irradiation are due to radiation induced volumetric decrease of the organ. Pancreas is indeed very sensitive to irradiation which is not known by radiation oncologists and should be considered as an organ at risk. Special care should be taken to reduce the radiation dose to the organ during radiation treatment planning for abdominal tumors.

## Methods

Patients with histologically proven nonmetastatic gastric cancer who had curative surgical resection were evaluated for adjuvant treatment and were included in this prospective study. Forty-nine patients presenting with serosal or adjacent visceral organ invasion (pT3, T4), or with involved lymph nodes were considered suitable for adjuvant treatment and these patients comprised the study group. Twenty-seven patients who did not need adjuvant treatment (pT1,pT2 and pN0) comprised the control group.

Adjuvant treatment plan in the study group was similar to the intergroup 0116 trial presented in 2001 by MacDonald et al. [14]. Patients received bolus 5-fluorouracil, one cycle before, two cycles concomitant with, and one cycle after radiation treatment. Radiation was delivered with either 6 or 15 MV photons by three-dimensional conformal technique including anastomosis, tumor bed and regional lymphatics similar to the technique described by Smalley et al. [15]. The radiation dose prescribed to planning target volume (PTV) constituted as mentioned above was 45 Gy in 25 fractions with 1.8 Gy fractions per day, 5 days per week for 5 weeks.

Informed consent was obtained from all the patients and the study was approved by the local ethics committee of the hospital.

Planning tomographies were obtained from all the patients in the study group for radiation planning purpose by either 3 or 5 mm slice thickness. Diognostic tomographies for all the patients in the study and control groups were obtained by 5 mm slice thickness.

During planning critical organs like liver, kidneys, spinal cord were contoured together with pancreas. Pancreas was contoured by an experienced radiologist on planning tomographies and mean pancreatic volume of the patients was measured as cm^3^. No caution was taken to decrease radiation dose to the pancreas since it was not considered as an organ at risk for radiotherapy planning. Most part of the pancreas was included within the PTV and the organ received almost the same dose as the PTV.

Radiation treatment was planned by conformal technique, and Eclipse treatment planning system (Version 10.0; Varian Medical Systems, Palo Alto, CA, USA) was utilized for dosimetric and volumetric measurements and for dose-volume histogram generations. For patients in the control group their first postoperative control abdominal tomographies were imported to planning system for pancreas contouring and volumetric measurements. For patients in the study group at least one year after radiotherapy, and for patients in the control group at least one year after surgery control abdominal tomographies obtained at follow-up were imported to the planning system for the same measurements. Mean pancreatic dose and the pancreas volume that received 20 Gy (V20), 30 Gy (V30), 40 Gy (V40) and 45 Gy (V45) were calculated for patients in the study group. Pancreas volumes measured at the beginning of the study and at least one year later were compared in each group itself and in between the two groups themselves.

Analysis was performed with the statistical package for the social sciences (SPSS 17.0). Within group significance was determined by the Wilcoxon signed rank test, between group differences by the Mann-Whitney and x^2^ tests. Results were expressed as mean ± SD. *P* value of less than 0,05 was considered to indicate statistical significance.

## Results

Patient charactheristics including T and N stages, and surgical resection type are summarized in Table 1.

**Table 1:** Patient charactheristics.

	Study group Radiotherapy (+) N:49	Control group Radiotherapy (−) N:27
Age (years)	56.16 ± 10.77	57 ± 11.69
Gender(F/M)	13/36	10/17
T Stage		
T1	–	7 (26%)
T2	6 (12%)	20 (74%)
T3	21 (43%)	–
T4	22 (45%)	–
N Stage		
N0	4 (8%)	27 (100%)
N1	10 (20%)	–
N2	15 (31%)	–
N3	20 (41%)	–
Surgical procedure		
Subtotal	22 (45%)	13 (48%)
Total	27 (55%)	14 (52%)

At the beginning of the study, mean pancreatic volume of the patients was 51,34 ± 20,33 cm^3^ (range; 18,04-97,47) in the study group and 50,12 ± 23,75 cm^3^ (range; 18,87-95,73) in the control group. The measured mean pancreatic volumes of both groups were similar (*p* = 0,63). Mean pancreatic volumes measured one year after radiotherapy for the study group, and one year after surgery for the control group were decreased. The measured volumes were 22,48 ± 10,53 cm^3^ (range; 6,27–49,55 cm^3^) for the study group, and 44,18 ± 23,08 cm^3^ (range; 14,8–91,3 cm^3^) for the control group. The difference in reductions in pancreatic volumes compared to the beginning values were statistically significant (*p* < 0,001 for both groups). While the pancreatic volume decreased by 56% in the study group, the decrease was only 12% in the control group. When the two groups were compared with each other with respect to pancreatic volume reductions at the end of the study, we found that the decrease in pancreas volume was significantly more pronounced in the irradiated group (22,48 ± 10,53 cm^3^) with respect to the control group (44,18 ± 23,08 cm^3^) (*p* < 0,001). Volume loss in pancreas was still statistically significant when the two groups were compared with respect to extent of surgery (total or subtotal gastrectomy) or when only pN0 or pT2 patients within each group were compared.

The mean pancreatic volumes at the beginning of the study and after one year for both groups are presented in Table 2.

**Table 2:** Mean pancreatic volumes, comparison of study group with control group

	Initial pancreas volume (cm^3^)	Pancreas volume after one year (cm^3^)	*p*
Study group	51,34 ± 20,33 (18,04-97,47)	22,48 ± 10,53 (6,27-49,55)	‹0,001
Control group	50,12 ± 23,75 (18,87-95,73)	44,18 ± 23,08 (14,8-91,3)	‹0,001
p	0,63	‹0,001	

Mean pancreatic radiation dose for patients in the study group was 4405,2 ± 486,9 cGy (range; 1983,4–4845 cGy). Pancreatic volume receiving 20 Gy, 30 Gy, 40 Gy and 45 Gy were 96,7%, 92,6%, 85,2 and 74% respectively. The maximum dose received by pancreas was 4829,4 ± 149,3 cGy (4289–5065 cGy), and the minumum dose was 2505,9 ± 1164,8 cGy (80,6–4466 cGy). Pancreas doses are summarized in Table 3.

**Table 3:** Pancreatic radiation doses in the study group patients

Mean Pancreatic Radiation Dose	4405,2 ± 486,9 (cGy)
Minimum Pancreatic Radiation Dose	2505,9 ± 1164,8 (cGy)
Maximum Pancreatic Radiation Dose	4829,4 ± 149,3 (cGy)
Pancreas V20	96,7% ± 10,5 (47,13–100)
Pancreas V30	92,6% ± 13,9 (36,3–100)
Pancreas V40	85,2% ± 20,6 (3,51–100)
Pancreas V45	74% ± 26,1 (0–99,7)
Pancreas D40	4621,9 ± 398,4 cGy (2607,8-4948,7)
Pancreas D50	4561,1 ± 507,6 cGy (1912–4937,5)
Pancreas D70	4325,6 ± 786,5 cGy (812,5-4893,2)
Pancreas D80	4118,8 ± 930,8 cGy (226,7-4781,7)
Pancreas D90	3733 ± 1036 cGy (127,2-4754,4)

Since there is no known dose restriction defined in the literature for the pancreas in contrast to the other organs like liver, kidneys and no special attention has been given to decrease the dose to the pancreas, the mean pancreatic radiation dose was high, and the organ received almost the same dose as the PTV.

## Discussion

Radiation induced late toxicity on pancreatic tissue is not an important concern and not studied extensively since pancreatic cancer patients treated with radiotherapy do not live long enough to explore the side effects of radiotherapy on this organ. The exact dose of radiation which causes 5% of the patients to have radiation induced pancreatic complications within 5 years is unknown. Pancreas has not been mentioned as an organ at risk, either in the quantitative analyses of normal tissue effects in the clinic report (QUANTEC), or in Emami’s late tissue toxicity report [1, 2].

Most research data related with radiation toxicity on pancreas comes from animal experiments and patients who were exposed to abdominal irradiation for curable diseases like lymphomas, childhood tumors, or for hematologic malignancies treated with total body irradiation [16–23]. However, these animal experiments and retrospective human studies are not enough to draw firm conclusions about radiation related late side effects on pancreas in order to define any safe dose or volume limits for the organ during radiation treatment planning.

With the implementation of adjuvant radiotherapy in management of gastric cancer after intergroup 0116 trial, abdominal irradiation became a standart treatment following surgery [14]. These patients who have now longer life span due to the adjuvant radiotherapy can give us the opportunity to study the late side effects of irradiation on pancreatic tissue. Late toxicity related to pancreatic exposure to radiation which has not been addressed prospectively before was recently studied in these patients both for endocrine and exocrine functions of the pancreas [12, 13].

In our previous prospective study, we found out that endocrine capacity of the organ was very sensitive to irradiation which demonstrated itself with decrease of beta-cell function after abdominal radiotherapy [12]. Another successive study in the same group of patients demonstrated exocrine functional loss of the pancreatic tissue after abdominal irradiation as well [13]. We think that these radiation sensitive endocrine and exocrine functional losses are due to radiation induced atrophy of the organ. In this prospective study we evaluated our hypothesis comparing a group of gastric cancer patients undergoing adjuvant abdominal radiotherapy with a group of patients who did not need it after curative gastric cancer resection. Both groups were operated either with total or subtotal gastrectomy with a similar percentage, and both groups had the same pancreatic volumes at the beginning of the study. One year after abdominal radiotherapy there was statistically significant decrease in pancreas volume of the irradiated patients (*p* < 0,001). Although both groups demonstrated decrease in the volume of the organ during the study period, the reduction rate in the irradiated group was 56%, whereas it was only 12% in the control group. This difference was statistically significant (*p* < 0,001). Volume loss in pancreas was still statistically significant when the groups were compared with respect to extend of surgery (total or subtotal gastrectomy) or when only pN0 or pT2 patients within each group were compared.

Volumetric decrease in certain organs such as salivary glands, kidneys and brain is a well-known effect of unavoidable radiation exposure during treatment of tumors neighbouring these critical tissues [3–7]. Late tissue toxicity as in the salivary glands, kidneys and brain is due to volumetric loss of the tissues after radiotherapy resulting in loss of organ function later. Pancreas has a very similar tissue structure and function to the salivary glands and it can be considered as the salivary gland of the gastrointestinal tract. Although radiation effect on salivary glands is very well known and studied extensively, this is not the case for pancreas. Salivary glands are the major organs at risk for head and neck radiotherapy planning. Although tolerance doses and dose constraints of salivary glands are well defined and determinant for final plan approval in radiation oncology practice, this is not the case for pancreas.

Animal experiments performed in seventies and eighties were very informative about the radiation sensitivity of the pancreas [20–23]. However, this information was not taken into consideration by radiation oncologists probably due to the short life span of pancreatic cancer patients. In one of these experiments, Pieroni et al. studied the effect of irradiation on pancreatic tissues of dogs [20]. They exposed the pancreas of the dogs to 40 Gy equivalent of radiation delivered in 6 weeks. Three months after irradiation there was significant decrease in pancreatic enzyme output (over 90%) together with diffuse interstitial fibrosis and marked reduction in acinar cells. Despite alteration in exocrine pancreatic function of the irradiated dogs, there was no evidence of diabetes, and no change in either fasting blood glucose or glucose tolerence tests of the dogs. Authors stated that islet cells were relatively resistant to irradiation at these dose levels compared to acinar cells. This animal experiment demonstrated that acinar cell damage was inevitable after exposure of the organ to fractionated 40 Gy radiotherapy [20]. Forty Gy or higher radiation doses are prescribed in the clinic for the treatment of upper abdominal malignancies as in the adjuvant treatment of gastric cancer.

Nacchiero et al. in another dog experiment examined simultaneously parotid and pancreatic gland secretions of dogs after exposure of these glands to 24 Gy of irradiation delivered in two weeks [21]. They noted marked reduction both in parotid and pancreatic gland secretions of the dogs within eight months. On histologic examination while there was diffuse insterstitial fibrosis and reduction of acinar cells in pancreatic glands of the dogs, these findings were absent in their parotid glands [21]. Heijmans et al. in another animal experiment studied the effect of intra-operative radiotherapy (IORT) on pancreas of dogs [22]. They tried to examine tolerance of the pancreatic tissue to IORT to define safe IORT doses for abdominal tumor treatment in humans. The authors noted significant reduction in serum insulin levels without overt diabetes at 30–35 Gy IORT doses. However, they found no meaningful change in the dogs irradiated at 25 Gy IORT dose level. They reported that IORT to the pancreas below 25 Gy was safe clinically [22]. Another IORT study performed in dogs by Ahmadu-Suka et al. demonstrated that there was atrophy of the gland, together with damage in pancreatic vasculature and ducts which resulted in eventual fibrosis of the organ [23]. Authors stated that exocrine pancreatic insufficiency and diabetes mellitus were possible potential late complications when IORT dose administered was above 25 Gy, and thus radiation dose received by pancreas during IORT should be tried to be kept below 25 Gy.

In parralel to the above-mentioned animal experiments, retrospective human studies demonstrated the sensitivity of the pancreas to radiation exposure in humans as well. Childhood cancer survivors and adults whose treatment included abdominal irradiation or total body irradiation have high prevalance of diabetes which becomes overt years after radiation exposure [16–19, 24–27]. The risk of diabetes increases as the child’s age of exposure to radiation decreases [24]. There is a dose-response relationship between radiation exposure of pancreas and the subsequent risk of diabetes [24]. De Vathaire et al. in a retrospective study that included children who were treated for solid tumors or lymphomas with abdominal radiotherapy as part of their treatment tried to calculate the radiation dose received by pancreas [24]. The authors created a dose calculation algorithm to measure the radiation dose by using children’s records and information about treatment machine and treatment details. They reported that the risk of diabetes increased as the radiation dose to the tail of pancreas increased. While the relative risk of diabetes was 11,5% in children who received 10 Gy or more to the tail of the pancreas, the risk increased with radiation doses above 20–29 Gy.

Radiation induced diabetes, a common observation in childhood cancer survivors, is also observed with a similar incidence in adults who are exposed to abdominal radiotherapy for different diseases. In a study originally planned to investigate the long-term cancer risk of abdominal radiotherapy in adults who were irradiated for peptic ulcer, the authors noticed high incidence of diabetes in the irradiated patients [28]. Radiation dose received by the pancreas was tried to be estimated from radiotherapy records of the patients and phantom measurements as in the De Vathaire et al. study mentioned above. The authors reported that relative risk of death due to diabetes increased with increased pancreatic radiation dose and found to be highest for 17–38 Gy dose levels [28].

All these animal and human data clearly demonstrate that pancreas is an organ at risk for radiation treatment. Acinar cells of the pancreas are much more sensitive to radiation than islet cells. While there is atrophy and loss of acinar cells after irradiation, islets of Langerhans are well preserved histologically but lose their functional capacities with diabetes mellitus risk later [18–23, 26–33]. Vascular damage is the most common cause of radiation toxicity. The irradiated pancreatic tissue shrinks in size and becomes extremely fibrotic with marked atrophy and volumetric loss of the organ. While animal experiments demonstrated sensitivity of exocrine function of the pancreas to radiation exposure which appeared early within one year, the human studies on the other hand demonstrated sensitivity of endocrine part of the pancreas which appeared years after radiation exposure of the pancreas [18–23, 26–33].

We think that pancreas has the same sensitivity to radiation as the salivary glands, especially the acinar cells of the organ which are responsible from exocrine function. High radiation sensitivity of acinar cells may be due to their rapid cell turnover as in the cells in early responding tissues. For preservation of exocrine function, the mean radiation dose to the pancreas should be kept as low as possible, and below 20–25 Gy delivered with classical fractionation. This is similar to the dose constraint defined for the parotid glands. On the other hand, islet cells of the pancreas responsible from endocrine function of the organ are probably more resistant to radiation than acinar cells. We think that islet cells have slow cell turnover and are comparable to the cells in late responding tissues. Pancreas as an organ at risk has similarity to the organs with parallel organization and hence can tolerate high radiation doses given to a limited volume except the tail part of the organ where the islet cells are located. Mean pancreatic radiation dose is the most important parameter that should be taken in to account during abdominal radiation treatment planning.

We hypothesize that for preservation of endocrine function after radiation exposure of the pancreas, mean radiation dose should be tried to be kept below 25 Gy. The latency periods for exocrine and endocrine functional losses are also different; observed early within one year for exocrine function, and observed very late for endocrine funtion [12, 13, 20–23]. This observation is important in the clinic for proposing early enzyme substitution for exocrine pancreatic insufficiency, and diet and life style changes for prevention of future diabetes risk.

We previously demonstrated in a prospective study that the radiation exposure of the pancreas results in functional loss of the organ [12]. In this successive study we demonstrated for the first time in the literature that, pancreas undergoes prominent atrophy after radiation exposure. Loss of pancreatic function results from its volumetric loss as observed in the salivary glands, kidneys and brain.

Pancreas should be considered as an organ at risk for radiation treatment planning. It should be contoured in the planning tomographies to create dose-volume histograms for the organ. The mean organ doses should be tried to be kept below 20–25 Gy for preservation of exocrine function, and below 25 Gy especially to the tail of pancreas for preservation of endocrine function. This is the first study paying attention to the pancreas as an organ at risk and defining safe mean radiation doses for the organ during abdominal radiotherapy plannings where pancreas is included within the radiation field.

## Conclusions

Pancreas has a very similar tissue structure and function to the salivary glands, and it can be considered as the salivary gland of the gastrointestinal tract. We think that the previously demonstrated endocrine and exocrine functional losses of the organ after abdominal irradiation are related to radiation induced volumetric loss of the pancreas as in the salivary glands. Special attention must be given to reduce the radiation dose to the organ during abdominal radiation treatment planning. Pancreas should be considered as an organ at risk and should be contoured in the planning tomographies to create its dose-volume histogram. Mean pancreatic radiation dose should be kept below 20–25 Gy to preserve the organ’s functions.

## Abbreviations

IORT: Intraoperative radiotherapy.
PTV: Planning target volume.
QUANTEC: Quantitative analyses of normal tissue effects in the clinic.
SPSS: Statistical package for the social sciences.
